# Antibacterial effect of chitosan from squid pens against *Porphyromonas gingivalis* bacteria

**Published:** 2019-04

**Authors:** Latief Mooduto, Dian Agustin Wahjuningrum, Agatha Prita A, Cecilia G. J. Lunardhi

**Affiliations:** Department of Conservative Dentistry, Faculty of Dental Medicine, Universitas Airlangga, Surabaya, Indonesia

**Keywords:** Chitosan, Squid pen, Antibacterial effect, *Porphyromonas gingivalis*

## Abstract

**Background and Objectives:**

Chitosan, a polysaccharide derived from squid pens – the squid waste, is gaining considerable interests in biomedical engineering due to the biodegradability, biocompatibility, nontoxicity, and antibacterial activity. It is necessary to eradicate the bacteria from root canal in endodontic treatment, including *Porphyromonas gingivalis. P. gingivalis* is one of the most prevalently found bacteria in root canals and its presence can cause endodontic treatment failure. This study was conducted to find the antibacterial effect of chitosan from squid pen against *P. gingivalis* at a certain concentration.

**Materials and Methods:**

Chitosan 1.5% (w/v) was diluted in several tubes. The lowest concentration with no bacterial growth was considered to have antibacterial activity against *P. gingivalis.*

**Results:**

There was no bacterial growth in nutrient agar media at the concentration of 10.75%.

**Conclusion:**

Chitosan that was made from squid pens has antibacterial activity against *P. gingivalis.*

## INTRODUCTION

Chitosan is a polysaccharide derived from chitin through deacetylation process (1). It is widely developed for various biomedical purposes such as tissue engineering, bone, nerves, skin, wound-healing, and burn wound treatment (2). The antibacterial activity of chitosan is considered to be able to affect wide-spectrum of bacteria (1).

In this study, the chitosan antibacterial test was performed against *Porphyromonas gingivalis*. *P. gingivalis* is an anaerobic Gram-negative bacteria with its cell membrane mainly composed of lipopolysaccharides (3). This bacteria is one of the dominant pathogenic bacteria involved in endodontic-perio lesions (4, 5).

## MATERIALS AND METHODS

### Chitosan production.

Chitosan production from squid pen was done in laboratory according to Chaussard and Domard method in Goy et al. (1). Squid pen with an average length of 30.4 cm in total of 10.7 grams that had been taken from the dorsal part of squid was dried using an oven at 50°C for 8 hours. Squid pen that has been dried was ground with a grinder to produce squid pen powder in size of 60 mesh. Shortly, the squid powder was then mixed with 10% NaOH and heated at 60°C with constant stirring at the rotary shaker at 125 rpm for 24 hours for deproteinization and then added into vacuum filtration to remove residual water. The obtained dried squid pen powder was washed to neutralizeits pH (pH = 7) then lyophilized using freeze drier into a powder called chitosan. In this study, we managed to obtain 1.765 gr of chitosan.

Chitosan suspension was prepared by mixing 1.5 gram of chitosan powder with 100 ml of 1% acetic acid to get 1.5% (w/v) chitosan suspension.

### Antibacterial activity test.

The antibacterial test of chitosan from squid pen was carried by dilution method. Based on previous research, it was found that concentrations with antibacterial activity potency were between 12.5% and 6.25%. In this study, 10 ml chitosan suspension was used for the concentration of 12.5% and then diluted four times to a concentration of 6.25% in 5 test tubes. Aliquot of 0.1 ml standardized suspension of *P. gingivalis* bacteria with 0.5 McFarland (1.5 × 10^8^ CFU/ml) was added to the test tube. Each tube was incubated in anaerobic condition in the incubator for 2 × 24 hours at 37°C. After that, the culture was spread into the nutrient media and incubated again for 24 hours to allow bacterial growth in order to do the calculation of colonies. If the number of colonies in the culture medium is less than 0.1% of the positive control, it means the material has antibacterial activity (6).

## RESULTS

The result of colony counting on each concentration is shown in Table 1. From the result shown in Table 1, the ability of chitosan in bacterial inhibition was increased as the concentration increase, it can be seen from the decrease of bacterial colony number.

**Table 1.** Results of colony counting in dilution method to test antibacterial activity against *Porphyromonas gingivalis*

	**N**	**Colonies mean (CFU)**	**Percentage**
Positive control	4	149.5	100%
Concentration of 12.5%	4	0	0%
Concentration of 10.75%	4	0	0%
Concentration of 9.25%	4	4,75	3, 16%
Concentration of 7.75%	4	9	5, 98%
Concentration of 6.25%	4	15,25	10, 13%

**Fig. 1.**
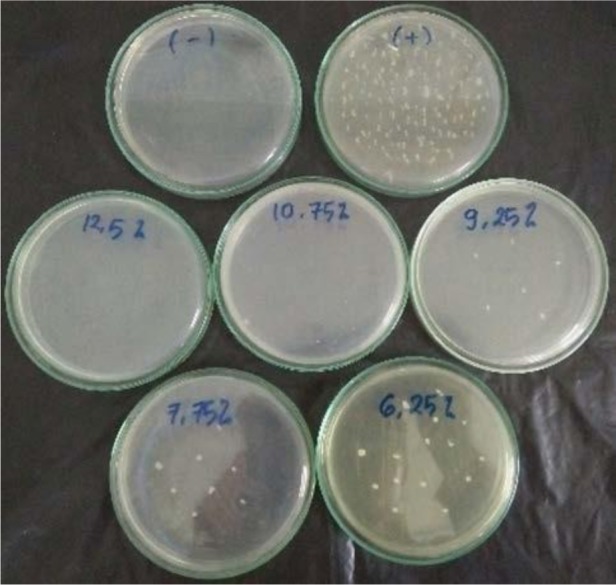
Cultivation of bacterial suspension in nutrient agar media for negative control (−), positive control (+), concentration of 12,5%, concentration of 10,75%, concentration of 9,25%, concentration of 7,75%, and concentration of 6,25%, to allow colony counting.

Based on the calculation of the colony number, it was found that bacterial growth at concentrations of 12.5% and 10.75% was 0% compared to positive control colonies or it can be concluded there was no bacterial growth. At the concentration of 9.25%, there are 3.16% bacterial colonies’ growth compared to positive control colonies. At the concentration of 7.75%, there are 5.98% bacterial colonies’ growth compared to positive control colonies, and at the concentration of 6.25%, there are 10.13% bacterial colonies’ growth compared to positive control colonies (Fig. 1).

## DISCUSSION

This experimental study aimed to find the antibacterial effect of chitosan from squid pen against *P. gingivalis. P. gingivalis* is known to be one of the etiological agents and key pathogens in the initiation of combined perio-endo lesions (7). *P. gingivalis* may survive in the host under anaerobic conditions as it may avoid host defense mechanisms by migrating across the basal membrane of the epithelial layer to invade connective tissue (5, 8).

The material used in this study is chitosan from squid pen which was suspected to have antibacterial effect against *P. gingivalis*. Based on previous research, it was found that concentrations with antibacterial potency were between 12.5% and 6.25%. Therefore, in this study, the concentration used was reduced to 12.5%, 10.75%, 9.25%, 7.75% and 6.25%. Chitosan used in this study is 1.5% (w/v). The solvent used to solve chitosan was 1% acetic acid because it effectively increases the inhibition effect of chitosan against bacterial growth (9).

The results of antibacterial test by the dilution method showed that the concentration of 10.75% was the minimum inhibition concentration (MIC). At the concentration of 9.25%, there were 3.16% bacterial colony growth compared to positive control colonies. At the concentration of 7.75%, there were 5.98% bacterial colony growth compared to positive control colonies, and at the concentration of 6.25%, there were 10.13% bacterial colony growth compared to positive control colonies.

From the results obtained, we found that chitosan from squid pen has antibacterial effect against *P. gingivalis*. Chitosan from squid pen is able to inhibit bacterial growth (bacteriostatic) and kill bacteria (bactericidal). The mechanism of inhibition of bacterial growth by chitosan is complicated and involves several factors. To date, there are three models proposed in connection with the mechanism of chitosan in inhibiting bacterial growth and killing bacteria. First, chitosan is able to inhibit the growth of *P. gingivalis* because chitosan has cationic properties (3). The cationic properties of chitosan are due to the amines, R-N (CH3)3+, so that chitosan interacts with the bacterial cell membrane which is negative. Strong electrostatic interaction between chitosan and bacterial cell membrane causes a change in the permeability of the bacterial membrane. As the permeability of the bacterial membrane alters, the transfer of material through the cell membrane is disrupted resulting in an osmotic pressure imbalance that triggers bacterial lysis. Because bacteria lysis, protein fluids and other intracellular materials will come out of the bacteria resulting in death (10, 11).

Second, chitosan can penetrate into the nucleus of bacterial cells and bind to bacterial DNA thus inhibiting the synthesis of mRNA and protein (9). Chitosan that binds to bacterial DNA can also interfere with the cell metabolic energy processes resulting in bacterial death (12). Third, chitosan works inhibiting and killing bacteria due to the metal chelating process. Chitosan molecules that surround the bacteria can form complexes with existing metals and inhibit the flow of important nutrients for bacteria (1).

From the three mechanisms proposed above, the electrostatic interactions that occur between chitosan and the outer membrane of bacteria are the most likely mechanisms to occur. According to Rafaat et al. (2018), chitosan has more effect on the outer membrane of bacteria because for the chitosan to penetrate up to the cell nucleus there are several layers of bacteria that must be penetrated and it requires certain particle size of chitosan (12).

In conclusion, Chitosan from squid pen has antibacterial activity against *P. gingivalis* bacteria at concentration of 10.75%.
